# Specialist palliative medicine physicians and nurses accuracy at predicting imminent death (within 72 hours): a short report

**DOI:** 10.1136/bmjspcare-2020-002224

**Published:** 2020-03-22

**Authors:** Nicola White, Fiona Reid, Victoria Vickerstaff, Priscilla Harries, Patrick Stone

**Affiliations:** 1 Marie Curie Palliative Care Research Department, University College London, London, UK; 2 School of Population Health & Environmental Sciences, King's College London, London, UK; 3 The Research Department of Primary Care and Population Health, University College London, London, UK; 4 Faculty of Health, Social Care and Education, Kingston University & St Georges, University of London, London, UK; 5 Department of Clinical Sciences, Brunel University, Uxbridge, UK

**Keywords:** prognosis, terminal care

## Abstract

**Objectives:**

Research suggests that clinicians are not very accurate at prognosticating in palliative care. The ‘horizon effect’ suggests that accuracy ought to be better when the survival of patients is shorter. The aim of this study was to determine the accuracy of specialist palliative care clinicians at identifying which patients are likely to die within 72 hours.

**Design:**

In a secondary data analysis of a prospective observational study, specialist palliative care doctors and nurses (in a hospice and a hospital palliative care team) provided survival predictions (yes/no/uncertain) about which patients would die within 72 hours.

**Results:**

Survival predictions were obtained for 49 patients. A prediction from a nurse was obtained for 37/49 patients. A prediction from a doctor was obtained for 46/49 patients. In total, 23 (47%)/49 patients actually died within 72 hours of assessment. Nurses accurately predicted the outcome in 27 (73%)/37 cases. Doctors accurately predicted the outcome in 30 (65%)/46 cases. When comparing predictions given on the same patients (27 [55%]/49), nurses were slightly better at recognising imminent death than doctors (positive predictive value (the proportion of patients who died when the clinician predicted death)=79% vs 60%, respectively). The difference in c-statistics (nurses 0.82 vs doctors 0.63) was not significant (p=0.13).

**Conclusion:**

Even when patients are in the terminal phase and close to death, clinicians are not very good at predicting how much longer they will survive. Further research is warranted to improve prognostication in this population.

## Background

The families of dying patients frequently want to know how much longer they have left to live,^1^ yet clinicians are not very accurate at predicting this,^2–4^ with accuracy ranging between 23% and 78%. A phenomenon known as the ‘horizon effect’ suggests that events occurring imminently ought to be more predictable.^2^ Thus, one might expect that clinicians should be better at recognising imminent death than at predicting longer-term survival. Accurate recognition of this phase can enable a ‘good death’; in which the patient’s final wishes can be achieved and harmful interventions can be ceased.^5–7^ However, recent reports^8 9^ have described that imminent death is not well recognised by clinicians and have noted a lack of quantitative research in this area.

Due to the wide variability of predicting survival, the evidence regarding the prognostic ability of different professional groups is inconsistent,^4^ and only a limited number of studies have specifically addressed the issue of prognostic accuracy when death is imminent.^10–12^

Understanding if there is a difference in prognostic accuracy by profession could inform future research about how to improve this clinical skill. This report is a secondary analysis of data from a larger programme of research designed to devise a method of testing clinicians’ prognostic accuracy.^13^ The purpose of this analysis is to:

1. Assess if specialist palliative care clinicians can accurately identify which patients will die in the following 72 hours.
2. Explore survival prediction performance by profession.

## Methods

This is a secondary analysis of data derived from a larger prospective, observational study.^14^ This report follows Standards for Reporting Diagnostic Accuracy Studies guidelines.

### Settings

Recruitment took place at two palliative care services in London, UK between January and October 2015: a North London hospice and a South London hospital.

### Participants

The participants in this study were specialist palliative care nurses and doctors at two sites (a hospice and a hospital) who were participating in a study to test clinicians’ prognostic accuracy.^13^

### Sample size

Fifty patients were recruited as part of the parent study. For each patient, a doctor and nurse involved in their care was asked for a survival prediction; making a total maximum possible of 50 predictions per profession.

### Consent procedure

The specialist palliative care teams at both site consented to participate in the parent programme of work. If they were unable or did not wish to provide a survival prediction, then no prognostic details were recorded. The parent study^13^ received approval from West Midlands—Coventry and Warwickshire Research Ethics Committee (May 2014, (14/WM/0121).

### Procedure

The specialist palliative care team at each site were responsible for identifying patients who were eligible for the parent study. Those who the team assessed and were identified as likely to die within 2 weeks were approached to participate. As part of the study assessments, a survival prediction (of death within 72 hours) was obtained from both a doctor and a nurse, where possible. The patient was reassessed after 7 days. If they died during this time, the date of death was documented.

### Main outcome

The main outcome was the prediction (yes/no) given by the clinician to the question about whether or not the patient was going to die within 72 hours. Clinicians were also given the option to say ‘I don’t know, or I am uncertain’ when they could not decide on the outcome.

### Analysis

The predictions of the specialist palliative care team were analysed by a professional group (nurse or doctor). The clinicians’ predictions and the actual survival outcomes of the patients are presented in a 2×2 table. Accuracy of prediction was assessed by sensitivity (the ability to recognise those who were dying), specificity (the ability to recognise those who were not dying), positive predictive value (PPV; the proportion of patients who died when the clinician predicted death) and negative predictive value (NPV; the proportion of patients who survived when the clinician predicted survival). The discrimination of the clinicians’ predictions was assessed using the c-statistic, also known as area under the curve. A c-statistic score of 0.5 indicates a model with poor predictive value. An increase in the score (to a maximum score of 1) indicates an increase in the level of clinician accuracy. ‘Uncertain’ predictions were not included in the analysis but were reported for transparency. The ‘roccomp’ command in STATA (V.16.0 was used for all analyses) was used to determine if the differences in c-statistic of the professions was significant, only in cases where there was a prediction from both professions.

## Results

The demographics of the patients recruited in to this study have been reported previously.^13^ A prediction was obtained for 49/50 patients recruited to the study (see online supplementary file 1). Nurses provided estimates for 37 (76%)/49 patients. Doctors provided estimates for 46 (94%)/49 patients. There was a prediction from both a doctor and a nurse in 27 (55%)/49 patients. In total, 23 (47%)/49 patients with a prediction died within 72 hours of assessment. Table 1 presents the accuracy of the predictions for doctors and nurses.

10.1136/bmjspcare-2020-002224.supp1 Supplementary data

**Table 1:** The accuracy of survival estimates by profession

	Total	N	Patient outcome		Results				
			Died	Survived	PPV	NPV	Sensitivity	Specificity	c-Statistic
					% (95% CI)				
**All estimates**									
Nurse	37								
Yes*		15	12	3	80 (52 to 96)	88 (64 to 99)	86 (57 to 98)	83 (59 to 96)	0.85 (0.72 to 0.98)
No†		17	2	15
Unsure‡		5	1	4					
Doctor	46								
Yes*		27	17	10	63 (42 to 81)	75 (48 to 93)	81 (58 to 95)	56 (32 to 76)	0.68 (0.54 to 0.81)
No†		16	4	12
Unsure‡		3	2	1					
**Patients with an estimate from each profession**	27								
Nurse									
Yes*		14	11	3	79 (49 to 95)	85 (55 to 98)	85 (55 to 98)	79 (49 to 95)	0.82 (0.67 to 0.97)
No†		13	2	11
Doctor	27								
Yes*		15	9	6	60 (32 to 84)	67 (35 to 90)	69 (39 to 91)	57 (29 to 82)	0.63 (0.44 to 0.82)
No†		12	4	8

*Yes, the patient will die within 72 hours.

†No, the patient will not die within 72 hours.

‡Unsure on survival (not included in the analysis of accuracy).

NPV, negative predictive value; PPV, positive predictive value.

### Nurse predictions

Nurses accurately predicted the outcome in 27 (73%)/37 cases. The nurses predicted that 15 (41%)/37 patients were going to die within 72 hours of the assessment: of those, 12 died (PPV=80%). They predicted that 17 (46%)/37 would survive and out of those, 15 survived (NPV=88%). They gave an ‘uncertain’ predictions in 5 (14%)/37 cases, one of whom died within 72 hours. The c-statistic for nurses was 0.85 (95% CI 0.72 to 0.98).

### Doctor predictions

Doctors accurately predicted the outcome in 30 (65%)/46 cases. The doctors predicted that 26 (57%)/46 patients were going to die within 72 hours of the assessment; of those, 17 died (PPV=65%). They predicted that 17 (37%)/46 would survive; of those, 13 survived (NPV=76%). The doctors gave an ‘uncertain’ prediction in three cases, two of whom died within 72 hours. The c-statistic for doctors was 0.68 (95% CI 0.54 to 0.81).

### Predictions by nurses and doctors for cases assessed by both

Exploring the 27 cases for whom there was a prediction available from both professions for comparison, the results for PPV were 79% (Nurse) vs 60% (Doctor), and for NPV were 85% (Nurse) vs 67% (Doctor; see table 1). The c-statistic for nurses predictions was 0.82 (95% CI 0.67 to 0.97), and for the doctors was 0.63 (95% CI 0.44 to 0.82). The difference between the c-statistic of the professions was not statistically different (p=0.13).

## Discussion

This study found that in the last 72 hours of life, clinicians’ predictions were accurate on between 65% and 73% of occasions, indicating that they were incorrect in their predictions on up to one in three occasions. In this study, nurses were slightly better than doctors at distinguishing between patients who were imminently dying and those who were not, a finding maintained after comparing only the cases that had a prediction from both professions; however the difference was not statistically significant.

Previous reviews have identified limited evidence about the reliability of predictions of imminent death.^8 15^ Our results are in keeping with some findings from previous studies,^10 12^ where it has been suggested that nursing staff are more accurate, although this is not a consistent trend. These findings warrant further research. If there is a difference between professionals then there may be something to learn from the differences between how nurses and doctors prognosticate, both locally and internationally.

This is one of only a limited number of prospective studies investigating the accuracy of predicting imminent death. As this was a secondary data analysis, the main limitation is the small number of prognostic estimates to compare (a maximum of 50 predictions per profession). In addition, the characteristics of the prognosticators (such as their age, experience or seniority) were not recorded. It is also important to note that the patients in the study were not a consecutive series of admissions to the service but were rather a selected group included as part of a larger study of prognostic accuracy.^13^ All patients were identified by the palliative care team as likely to die within 2 weeks. We do not have data on those who were not referred to the palliative care team or those patients whom the team did not feel were going to die within the next 2 weeks.

## Conclusion

The study findings indicate that even when predicting imminent death (72 hours), clinicians were inaccurate up to 1 in 3 of their predictions. Nurses were slightly better at recognising imminent death but a larger scale study would be required to explore this.
