# Supplementary figures and images for: Specialist palliative medicine physicians and nurses accuracy at predicting imminent death (within 72 hours): a short report
Source: BMJ Support Palliat Care. 2020 Mar 22;10(2):209–12. doi: 10.1136/bmjspcare-2020-002224 (PMC7286035; doi:10.1136/bmjspcare-2020-002224)

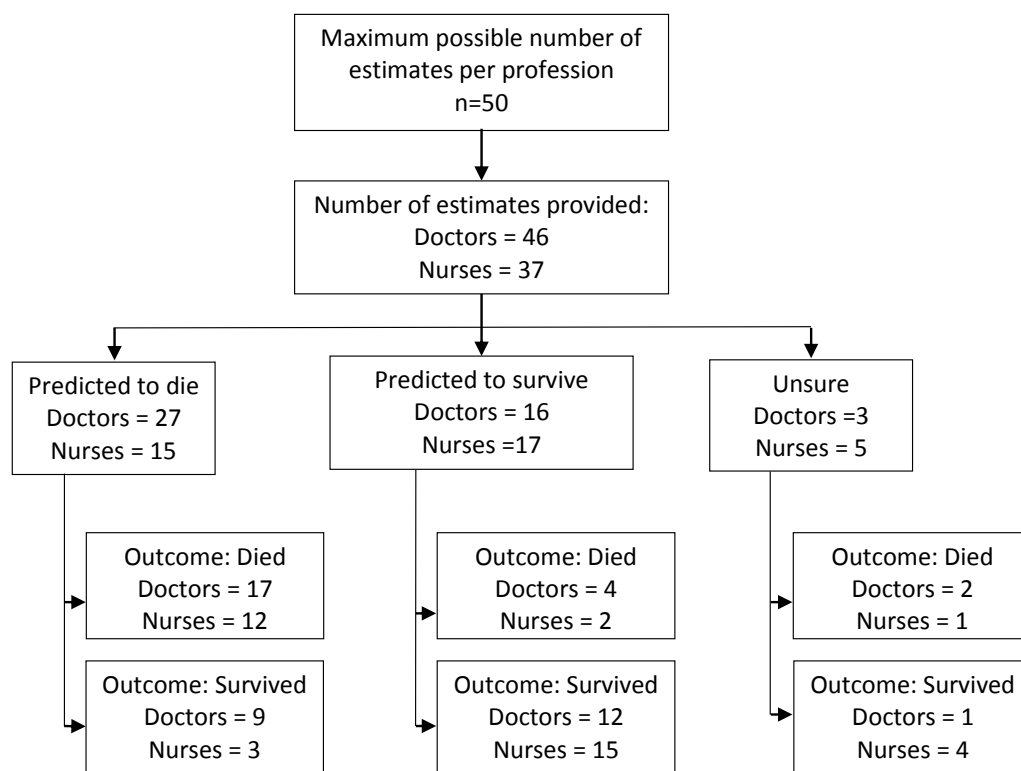

Supplementary File 1 STARD Study Flowchart

Supplement: Supplementary data [file bmjspcare-2020-002224supp001.pdf]
